# Diminished schwann cell repair responses play a role in delayed diabetes-associated wound healing

**DOI:** 10.3389/fphys.2022.814754

**Published:** 2022-12-22

**Authors:** Shaolong Zhou, Lingling Wan, Xu Liu, Delin Hu, Feng Lu, Xihang Chen, Fangguo Liang

**Affiliations:** ^1^ Aesthetic Medical School, Yichun University, Yichun, China; ^2^ School of Chemical and Biological Engineering, Yichun University, Yichun, China; ^3^ Department of Plastic and Cosmetic Surgery, Nanfang Hospital, Southern Medical University, Guangzhou, China

**Keywords:** diabetes, Schwann cell, de-differentiation, TGF-β, TIMP1, wound healing

## Abstract

Diabetes mellitus is the most common metabolic disease associated with impaired wound healing. Recently, Schwann cells (SCs), the glia of the peripheral nervous system, have been suggested to accelerate normal skin wound healing. However, the roles of SCs in diabetic wound healing are not fully understood. In this study, Full-thickness wounds were made in the dorsal skin of C57/B6 mice and *db/db* (diabetic) mice. Tissue samples were collected at different time points, and immunohistochemical and immunofluorescence analyses were performed to detect markers of de-differentiated SCs, including myelin basic protein, Sox 10, p75, c-Jun, and Ki67. In addition, *in vitro* experiments were performed using rat SC (RSC96) and murine fibroblast (L929) cell lines to examine the effects of high glucose conditions (50 mM) on the de-differentiation of SCs and the paracrine effects of SCs on myofibroblast formation. Here, we found that, compared with that in normal mice, wound healing was delayed and SCs failed to rapidly activate a repair program after skin wound injury in diabetic mice. Furthermore, we found that SCs from diabetic mice displayed functional impairments in cell de-differentiation, cell-cycle re-entry, and cell migration. *In vitro*, hyperglycemia impaired RSC 96 cell de-differentiation, cell-cycle re-entry, and cell migration, as well as their paracrine effects on myofibroblast formation, including the secretion of TGF-β and Timp1. These results suggest that delayed wound healing in diabetes is due in part to a diminished SC repair response and attenuated paracrine effects on myofibroblast formation.

## Introduction

### Background

Diabetes mellitus (DM) is the most common metabolic disease associated with impaired wound healing and is a worldwide health problem affecting approximately 9.3% of the global population. The prevalence of DM is expected to rise by 25% in 2030 (Saeedi et al., 2019).

Skin wound healing is a complicated and dynamic biological process that involves inflammation, cell proliferation, cell migration, angiogenesis, and re-epithelialization (Eming et al., 2014). DM-associated impairment of wound healing is accompanied by denervation-delayed wound contraction, altered fibroblast proliferation, delayed re-epithelialization, and reduced granulation tissue development (Nowak et al., 2021). Recently, the peripheral nervous system has been implicated in the promotion of wound healing (Silva et al., 2018). Peripheral nerve-associated Schwann cells (SCs) are able to promote the repair and regeneration of multiple tissue types, in addition to that of peripheral nervous system axons (Carr and Johnston, 2017). Innervation promotes the axonal sprouting of neurons and the associated secretion of growth factors in the wound bed upon injury (Barker et al., 2006). Upon denervation, SCs from peripheral nerves promptly de-differentiate into “repair” SCs that express high levels of the neurotrophin receptor p75 and low levels of myelin basic protein (MBP) (Hao, Tashiro, Hasegawa, Sato, Kobayashi, Tando, et al.). These repair SCs from disrupted peripheral nerves contribute to dermal wound healing by inducing the appearance of myofibroblasts at the wound site *via* TGF-β signaling. Genetic ablation of SCs delays wound contraction and closure, decreases myofibroblast formation, and impairs skin re-epithelization after injury (Parfejevs et al., 2018).

Compared with those in healthy controls, wounds in diabetic individuals display reduced levels of re-epithelialization and granulation tissue, poor vascularization, diminished dermal innervation, decreased numbers of superficial dermal SCs, and slower SC migration at the axotomy site (Ebenezer et al., 2011). Given the roles of SCs in wound healing, a lack of dermal SCs could contribute to impaired wound healing in DM patients. Notably, diabetes is associated with SC dysfunction, including increased apoptosis and abnormal de-differentiation (Hao, Tashiro, Hasegawa, Sato, Kobayashi, Tando, et al.; Zhu et al., 2018). However, it is unclear whether SCs do indeed contribute to the repair of diabetic wounds. A better understanding of the roles of SCs and their repair responses is essential to uncover the role of nerve plasticity in diabetic wound repair.

In this study, we examined the repair responses of SCs during diabetic wound healing. We found that diabetic SCs fail to rapidly activate a transcriptional repair program after injury and exhibit impaired de-differentiation, cell-cycle re-entry, and cell wound bed migration. Moreover, reduced secretion of the paracrine factors TGF-β and tissue inhibitor of matrix metalloproteinase 1(Timp1) by SCs in hyperglycemic conditions resulted in diminished fibroblast activation.

## Materials and methods

### Animals

All animal experiments were approved by the Nanfang Hospital Animal Ethics Committee Laboratory and were conducted according to the guidelines of the National Health and Medical Research Council of China. Male 8-weeks-old C57/BL6 mice and male 15-weeks-old BKS Cg-m^+/+^Lepr^*db*^/J (*db/db*) mice were obtained from Southern Medical University (Guangzhou, China). C57/BL6 mice and db/db mice were bred under standard conditions, and they were used for experiments at the same age (16-week-old). The db/m mice were commonly used as controls for db/db mice. However, C57/BL6 mice were also used as controls for db/db mice in skin wound healing (Maschalidi et al., 2022). Thus, we used C57/BL6 mice as controls for db/db mice in this study.

### Experimental wound model

**FIGURE 1:**
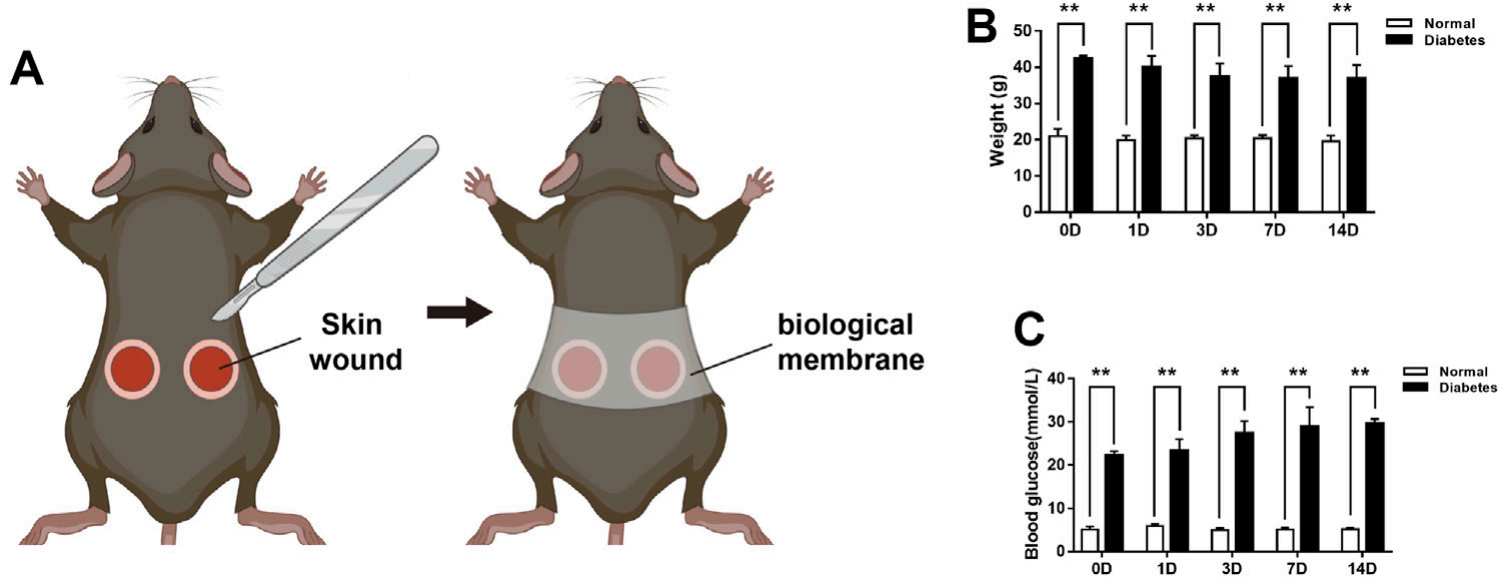
The experimental mice model and body weights and blood glucose levels of mice during wound healing **(A)** The wound healing model. Measurement of the mice body weights **(B)** and blood glucose levels **(C)** at days 0, 1, 3, 7, and 14 post-wounding. Data are presented as mean ± SD, **p < .01, *n* = 6/group, two-way ANOVA, Bonferroni’s post hoc test.

Following a protocol approved by the Nanfang Hospital Animal Ethics Committee Laboratory, general anesthesia of the mice was induced by 5% isoflurane in 100% O_2_ and was subsequently maintained using 3% isoflurane. Prior to surgery, the backs of the mice were shaved, cleaned thoroughly, and disinfected using antibacterial soap and 75% EtOH. Two circular full-thickness excisional wounds (6 mm diameter) were generated on each side of the lower back skin of each animal. For the wound model without contraction, a biological membrane (NPWT-1, negative pressure wound therapy kit; China) was glued to the surface of the wound with adhesive dressings before contraction (Yao et al., 2014; Wang et al., 2017). Due to the large amount of tissue required for the pathology examination, two wounds were performed per animal in this study, and the average of the two wounds was assessed per animal (Figure 1A). Following surgery, according to the guidelines, meloxicam (5 mg/kg) was used subcutaneously in the loose skin at the base of the neck in mice, and mice were placed on a warming pad (37°C) until they fully recovered from surgery and then recaged. Subsequently, the mice were housed in the institutional animal facility and were sacrificed 0, 1, 3, 7, or 14 days post-wounding (*n* = 6 per time point per group).

### Macroscopic wound size measurement

The wound areas were quantified using pictures taken from days 0–14 post-surgery.

### Histological analysis of murine skin samples

Murine skin tissue samples were embedded in paraffin, sectioned, and then stained with H&E or Masson’s trichome at different time points, according to routine procedures. For immunofluorescence analyses, skin tissue sections were incubated with the following primary antibodies: rat anti-mouse α-smooth muscle actin (α-SMA; 1:100; Abcam, Cambridge, United Kingdom), rat anti-mouse p75 (1:50, Abcam), rabbit anti-mouse MBP (1:1000, Abcam), rabbit anti-mouse SOX10 (1:100, Abcam), rabbit anti-mouse p75 (1:100, Abcam), rat anti-Ki67 (1:100, Abcam), and rabbit anti-mouse c-Jun (1:100, Abcam). Images were captured using a fluorescence microscope (IX71FL, Olympus), and the percentage of p75^high^ MBP^low^ NBs relative to total NBs, the percentage of p75^+^/SOX10^+^cells relative to total SOX10^+^cells were analyzed. And the numbers of p75^+^/Ki67^+^ cells and c-Jun+/Ki67^+^ cells in different groups were counted. The folded areas in all of these immunohistochemistry images have been excluded from quantification in this manuscript. And non-specific p75 stainings in the image’s folded tissue were also excluded from quantification in this manuscript.

### Cells and cell culture

Rat SCs (RSC96, ATCC^Ⓡ^CRL-2765™) and mouse skin fibroblasts (L929 cells) were cultured in DMEM containing 10% fetal bovine serum, 1% penicillin, and 1% streptomycin.

To explore the effects of high glucose, RSC96 cells were divided into two groups: normal glucose (5.5 mmol/L glucose) and high glucose (50 mmol/L glucose). After cell-cycle synchronization by serum deprivation, the cells were treated with the indicated concentrations of glucose for 48 h, and then cell biologic activities were detected.

To explore the effects of high glucose and TGF-β1 (Solarbio, Beijing, China), L929 cells were divided into three groups: normal glucose, high glucose and high glucose added with TGF-β1. After cell-cycle synchronization by serum deprivation, the cells were treated with normal glucose (5.5 mmol/L glucose), high glucose (50 mmol/L glucose) or high glucose added with TGF-β1 (10 ng/ml) for 24 h, and then cell biologic activities were detected.

To explore the paracrine effect of RSC96 cells grown under normal glucose (5.5 mmol/L glucose) condition. L929 cells grown under the same normal glucose condition (5.5 mmol/L glucose) were treated with RSC96 cell culture supernates from normal glucose group at 10% concentration for 24 h. As a control, L929 cells were cultured alone under normal glucose condition for 24 h. Subsequently, cell biologic activities were detected.

To explore the paracrine effect of RSC96 cells grown under high glucose (50 mmol/L glucose) condition. L929 cells grown under the same high glucose condition were treated with RSC96 cell culture supernates from high glucose group at 10% concentration for 24 h. As a control, L929 cells were cultured alone under high glucose condition for 24 h. Subsequently, cell biologic activities were detected.

### Cell migration assay

RSC96 cells were seeded into 6-well plates at a density of 2 × 10^5^ cells per well and were incubated under normal glucose (5.5 mmol/L glucose) or high glucose (50 mmol/L glucose) conditions for 48 h. Subsequently, a pipette tip was used to scratch the cell layer, and the migration area was calculated 36 h later.

L929 cells were seeded into 6-well plates at a density of 2 × 10^5^ cells per well and were incubated under normal glucose (5.5 mmol/L glucose), high glucose (50 mmol/L glucose) or high glucose added with TGF-β1 (10 ng/ml) conditions for 48 h. Subsequently, the cell layer was scratched using a pipette tip, and the migration area was calculated 24 h later using a Leica inverted microscope.

L929 cells were seeded into 6-well plates at a density of 2 × 10^5^ cells per well and were incubated with or without RSC96 cell culture supernates for 48 h. Subsequently, the cell layer was scratched using a pipette tip, and the migration area was calculated 24 h later using a Leica inverted microscope.

### Immunofluorescence analyses

RSC96 cells were fixed with 4% paraformaldehyde, permeabilized with phosphate-buffered saline (PBS) containing 0.1% Triton X-100, blocked with PBS containing 0.1% Tween and 5% goat serum, and then stained with rabbit anti-rat p75 (1:100, Abcam), rabbit anti-rat Ki67 (1:100, Abcam), and rabbit anti-rat c-Jun (1:100, Abcam) primary antibodies. The coverslips were then sequentially labeled with species-specific fluorochrome-conjugated secondary antibodies and DAPI (Sigma-Aldrich, St. Louis, MO, United States). Samples were visualized using a fluorescence microscope (Olympus). The numbers of p75^+^ cells, Ki67^+^ cells, and c-Jun^+^ cells were counted at ×400 original magnification.

L929 cells were fixed with 4% paraformaldehyde, permeabilized with PBS containing 0.1% Triton X-100, blocked with PBS containing 0.1% Tween and 5% goat serum, and then stained with rabbit anti-mouse α-SMA (1:100, Abcam) and rabbit anti-mouse Ki67 (1:100, Abcam) primary antibodies. The coverslips were then sequentially labeled with species-specific fluorochrome-conjugated secondary antibodies and DAPI (Sigma-Aldrich). Samples were visualized using a fluorescence microscope (Olympus). The numbers of α-SMA^+^ and Ki67^+^ cells were counted at ×400 original magnification.

### Quantitative PCR

Total RNA was isolated from L929 cells incubated under different glucose conditions for 12 h, and cDNA was synthesized using oligo (dT) primers and reverse transcriptase (Wako Pure Chemicals Industries, Osaka, Japan). Quantitative PCR was performed using SYBR Premix ExTaq II reagent and a DICE thermal cycler (Takara Bio, Inc., Tokyo, Japan), following the manufacturer’s instructions. *Gapdh* expression served as an internal control. The primers for *Tgfb1*, *Pdgfb*, *Timp1*, and *Gapdh* were as follows: *Tgfb1* F, GCA​ACA​ATT​CCT​GGC​GTT​AC; *Tgfb1* R, GTA​TTC​CGT​CTC​CTT​GGT​TCA​G; *Pdgfb* F, GAA​TAC​TTT​CAG​GCA​GGC​TAG​G; *Pdgfb* R, TAA​AGG​GAC​AGG​GAG​AGA​TGA​G; *Timp1* F, TGC​AAA​CTG​GAG​AGT​GAC​AG; *Timp1* R, GTA​TTG​CCA​GGT​GCA​CAA​ATC; *Gapdh* F, GGA​GAA​ACC​TGC​CAA​GTA​TGA; and *Gapdh* R, TTG​AAG​TCA​CAG​GAG​ACA​ACC.

### Measurement of TGF-β1 Levels *in vitro*.

The total TGF-β1 was measured using a Mouse TGF-β1 ELISA KIT (Solarbio) according to the manufacturer’s instructions.

### Statistical analysis

Data were analyzed using GraphPad Prism statistical software (GraphPad Software, Inc., La Jolla, CA, United States) and are presented as the mean ± standard deviation. Independent sample *t*-tests, one-way analyses of variance with Tukey’s *post hoc* tests, or two-way analyses of variance with Bonferroni’s *post hoc* tests were performed as appropriate. *p* < .05 was considered statistically significant.

## Results

### Diabetes delays cutaneous wound healing

Excisional wounds were inflicted on the backs of anesthetized normal (C57/BL6) and diabetic [BKS Cg-m^+/+^Lepr^*db*^/J (*db/db*)] mice, and the wound areas were measured macroscopically at 0, 1, 3, 7, and 14 days (D) post-wounding. Body weight were examined in *db/db* and normal mice during wound healing. As shown in Figure 1B, body weights were significantly higher in *db/db* mice compared to normal mice at different time points during wound healing. And the baseline glucose levels were 5.05 ± .73 mmol/L in the normal mice group and 22.27 ± .91 mmol/L in the *db/db* mice group. At 14 days post-wounding, the blood glucose levels were 5.12 ± .39 and 29.55 ± 1.16 mmol/L in the two groups respectively (Figure 1C).

**FIGURE 2:**
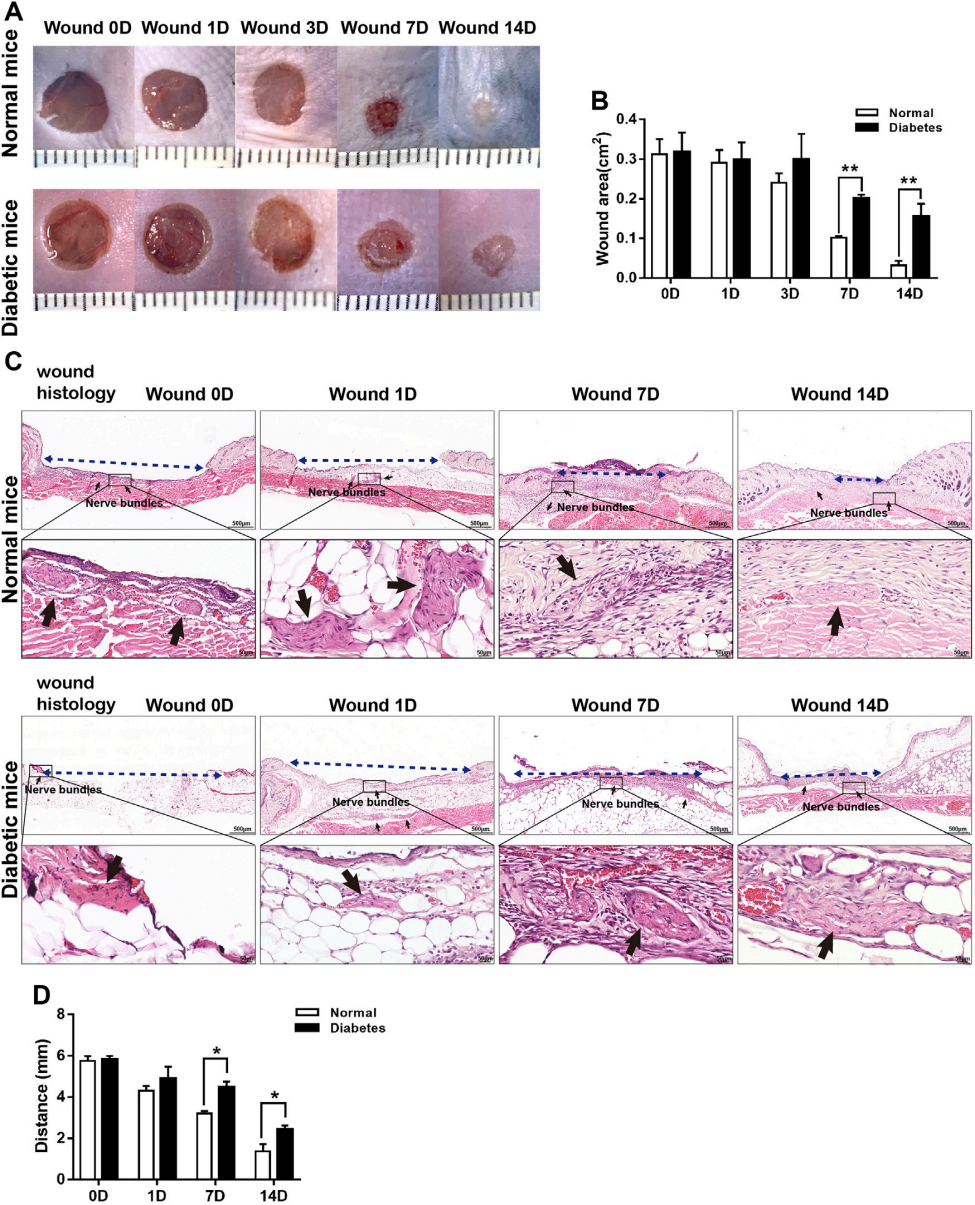
Diabetes delays cutaneous wound healing. **(A)** Representative macroscopic illustrations of normal and diabetic wounds on the backs of mice. **(B)** Measurement of the wound areas at days 0, 1, 3, 7, and 14 post-wounding. **(C)** H&E-stained sections of normal and diabetic wounds at days 0, 1, 7, and 14 post-wounding. The blue dotted lines represent wound contraction (distance between the wound border hair follicles), and the black arrows show the nerve bundles. **(D)** Measurement of wound contraction at days 1, 7, and 14 post-wounding. Data are presented as mean ± SD, **p < .01 and*p < .05, *n* = 6/group, two-way ANOVA, Bonferroni’s post hoc test.

The mean wound sizes in the normal and diabetic groups differed significantly at D7 (10.17 ± .42 vs. 20.10 ± .90 mm^2^, respectively) and D14 (3.21 ± 1.14 vs. 15.47 ± 3.32 mm^2^, respectively) (Figures 2A, B). Wound closure was also delayed in diabetic mice compared with normal mice (Figures 2C, D).

**FIGURE 3:**
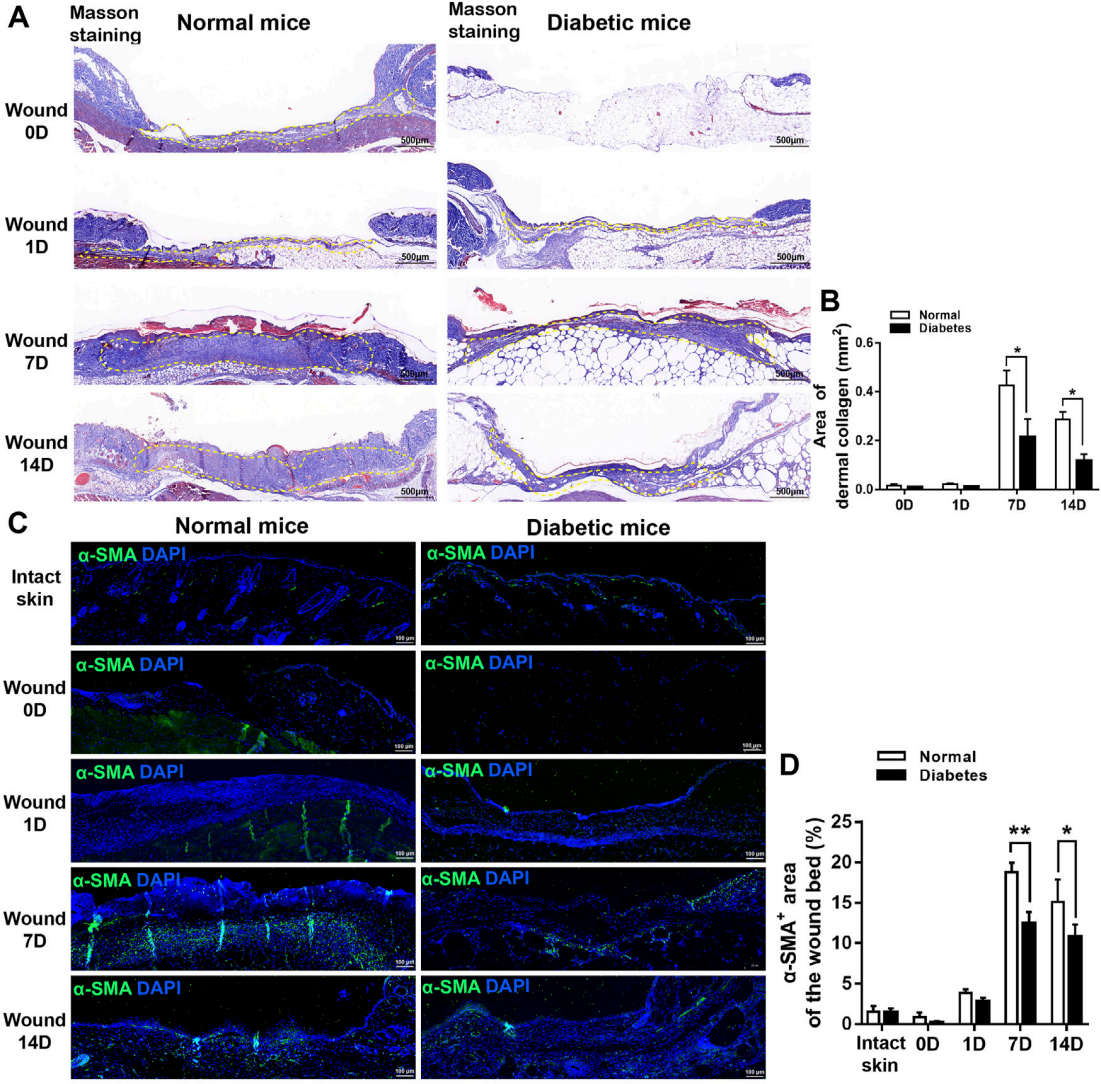
Diabetes impairs myofibroblast formation during cutaneous wound healing. **(A)** Representative images of Masson’s trichrome-stained sections of normal and diabetic wounds. **(B)** Measurement of the area of dermal collagen at days 0, 1, 7, and 14 post-wounding. The yellow dotted lines show the dermal collagen. **(C)** Representative immunofluorescence analyses of α-SMA expression in sections of normal and diabetic wounds. **(D)** Measurement of the α-SMA^+^ area in intact skin and in the wound bed at days 0, 1, 7, and 14 post-wounding. Data are presented as mean ± SD, **p < .01 and*p < .05, *n* = 6/group, two-way ANOVA, Bonferroni’s post hoc test.

In Masson’s trichrome-stained sections (Figures 3A, B), the mean area percentages of dermal collagen in the normal group were 0.43% ± 0.06%, and 0.29% ± 0.03% on D7 and D14, respectively. Collagen deposition was reduced significantly in the diabetic group at both time points (0.22 ± 0.07% and 0.12 ± 0.03% on D7 and D14, respectively; *p* < .05 vs. normal group).

Next, we examined the expression of α-SMA, a marker of myofibroblasts that plays a role in wound contraction, at the wound sites in normal and diabetic mice. The area of α-SMA expression was significantly lower in the diabetic group than in the normal group on D7 (18.79 ± 1.17% vs. 12.43 ± 1.45% of the total wound bed area, respectively) and D14 (15.13 ± 2.76% vs. 10.82 ± 1.50%, respectively) (Figures 3C, D). Overall, these findings indicate that diabetes delays wound healing in mice.

### Diabetic wounds display impaired SC de-differentiation

**FIGURE 4:**
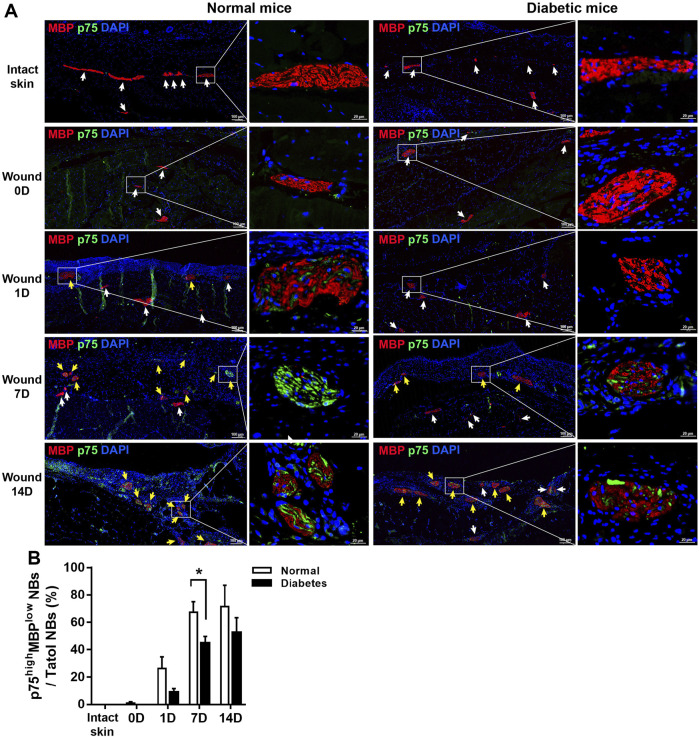
Diabetic SCs display delayed de-differentiation during cutaneous wound healing. **(A)** Representative immunofluorescence analyses of p75 (green) and MBP (red) in normal and diabetic mice in intact skin and in the wound bed at days 0, 1, 7, and 14 post-wounding. The white arrows represent the NBs displayed barely expression of p75 and high levels of MBP, the yellow arrows show the NBs displayed high levels of p75 and low levels of MBP. **(B)** Quantification of the percentage of p75^high^ MBP^low^ NBs relative to total NBs in normal and diabetic mice in intact skin and in the wound bed at days 0, 1, 7, and 14 post-wounding. Data are presented as mean ± SD, *p < .05, *n* = 6/group, two-way ANOVA, Bonferroni’s post hoc test.

Skin is a densely innervated organ, and peripheral nerve bundles (NBs) were visible during full-thickness excisional wound healing in both the normal and diabetic groups (Figure 2C). SCs in wound bed were supposed to undergo a rapid reprogramming process, dedifferentiating into non-myelinating repair cells that secrete proregenerative factors. We next directly addressed if diabetes affected SCs function and repair responses after skin wound. As showed in Figure 4A, in normal mice, the level of MBP was down-regulated as early as D1 post-wounding, and p75 expression, an induced marker for the repair cell phenotype, in the NBs was upregulated from D1 to D14 (Figures 4A, B), suggesting de-differentiation of SCs across this time period. Notably, p75^+^ SCs appeared to have disseminated from the disrupted nerves and migrated into the wound bed at D7 in normal mice. However, the expression of p75 was delayed in diabetic SCs after skin wound compared to normal controls. SCs failed to de-differentiate as early as D1, and p75 expression was low at this time point. The percentage of NBs displayed high levels of p75 protein and weak expressions of MBP in diabetic wound bed was significantly lower than that in normal group at D7 (Figure 4B).

**FIGURE 5:**
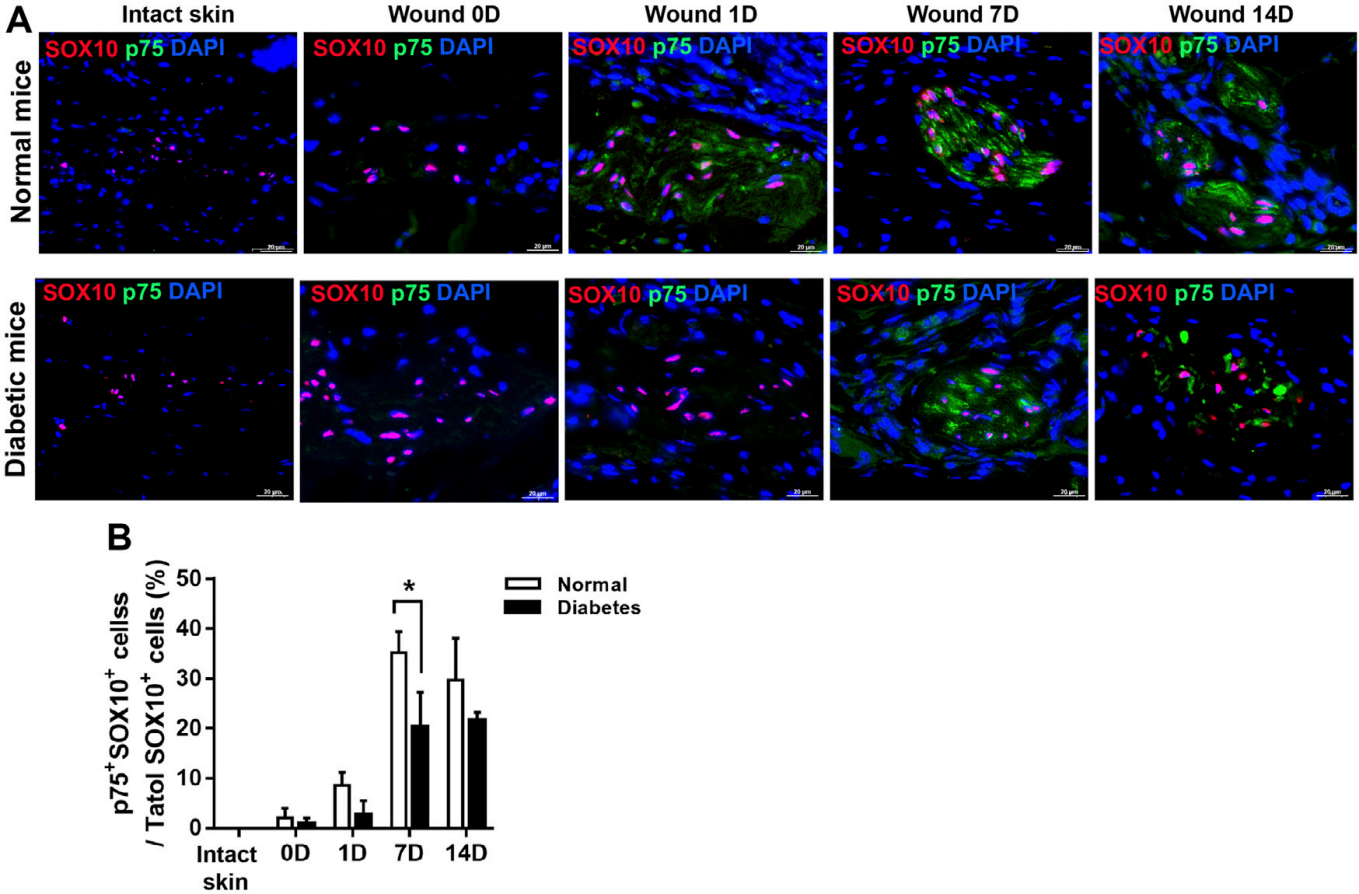
Diabetic wounds display reduced SC de-differentiation during cutaneous wound healing. **(A)** Representative immunofluorescence analyses of p75 (green) and SOX10 (red) in normal and diabetic mice at days 0, 1, 7, and 14 post-wounding. **(B)** The percentages of p75^+^SOX10^+^SCs in normal and diabetic mice in intact skin and in the wound bed at days 0, 1, 7, and 14 post-wounding. Data are presented as mean ± SD, *p < .05, *n* = 6/group, two-way ANOVA, Bonferroni’s post hoc test.

In addition, in normal wound bed, 8%, 35%, and 29% of SOX10^+^ SCs were positive for p75 at D 1, 7, and 14 respectively. SOX10 is an SC marker constitutively present in intact and dedifferentiated SCs. In diabetic wound, however, p75 reactivity was observed in only 2%, 20%, and 21% of SOX10 SCs at D 1, 7, and 14 respectively. These results show that skin injury promoted SCs de-differentiation in both normal and diabetic mice; however, cell de-differentiation was delayed, and cell migration was impaired in diabetic SCs compared with control SCs (Figures 5A, B). Thus this delay of SCs de-differentiation may lead to the delay of wound healing in diabetic mice.

### Diabetic wounds display impaired SC proliferation

**FIGURE 6:**
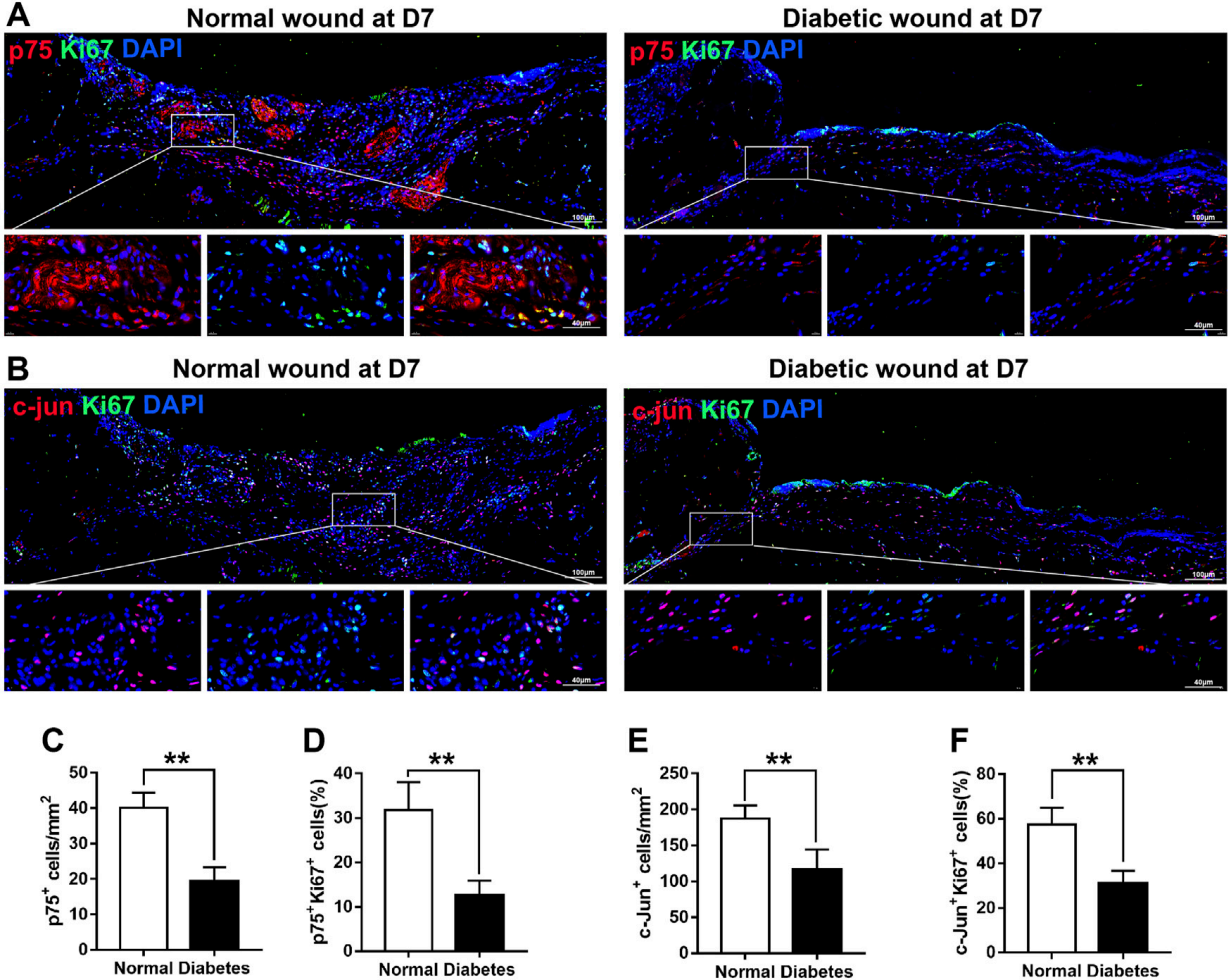
Diabetic wounds display reduced SC proliferation. **(A)** Representative immunofluorescence analyses of p75 (red) and Ki67 (green) in normal and diabetic mice at day 7 post-wounding. **(B)** Representative immunofluorescence analyses of c-Jun (red) and Ki67 (green) in normal and diabetic mice at day 7 post-wounding. **(C–F)** The numbers or percentages of p75^+^ cells **(C)**, p75^+^/Ki67^+^ cells **(D)**, c-Jun^+^ cells **(E)**, and c-Jun^+^/Ki67^+^ cells **(F)** in normal and diabetic mice at day 7 post-wounding. Data are presented as mean ± SD, **p < .01, *n* = 6/group, Student’s *t*-test.

At D7 after skin injury, the number of p75^+^ cells in the wound area of diabetic mice (19.20 ± 4.09 per mm^2^) was significantly lower than that in the wound area of normal mice (40.20 ± 4.21 per mm^2^) (Figures 6A, C). In addition, at D7, the number of Ki67^+^ and p75^+^ cells was lower in the diabetic group than in the normal group (12.6% ± 3.29% vs. 31.80% ± 6.23%, respectively; Figures 6A, D), indicating a reduced proliferation rate of de-differentiated SCs in the diabetic mice.

Notably, post-wounding expression of c-Jun, a marker associated with SC plasticity and de-differentiation, was lower in diabetic SCs than in normal SCs at D7 (187.60 ± 17.95 vs. 116.20 ± 28.28 mm^2^, respectively; Figures 6B, E). In normal animals, 57% of c-Jun^+^ cells were positive for Ki67 at D7 after skin injury; however, in diabetic animals, Ki67 reactivity was observed in only 31% of c-Jun^+^ cells (Figures 6B, F). Overall, these findings indicate that diabetic SCs fail to rapidly activate a transcriptional repair program after skin injury, which may lead to delayed wound contraction.

### High glucose reduces the viability and de-differentiation of SCs

**FIGURE 7:**
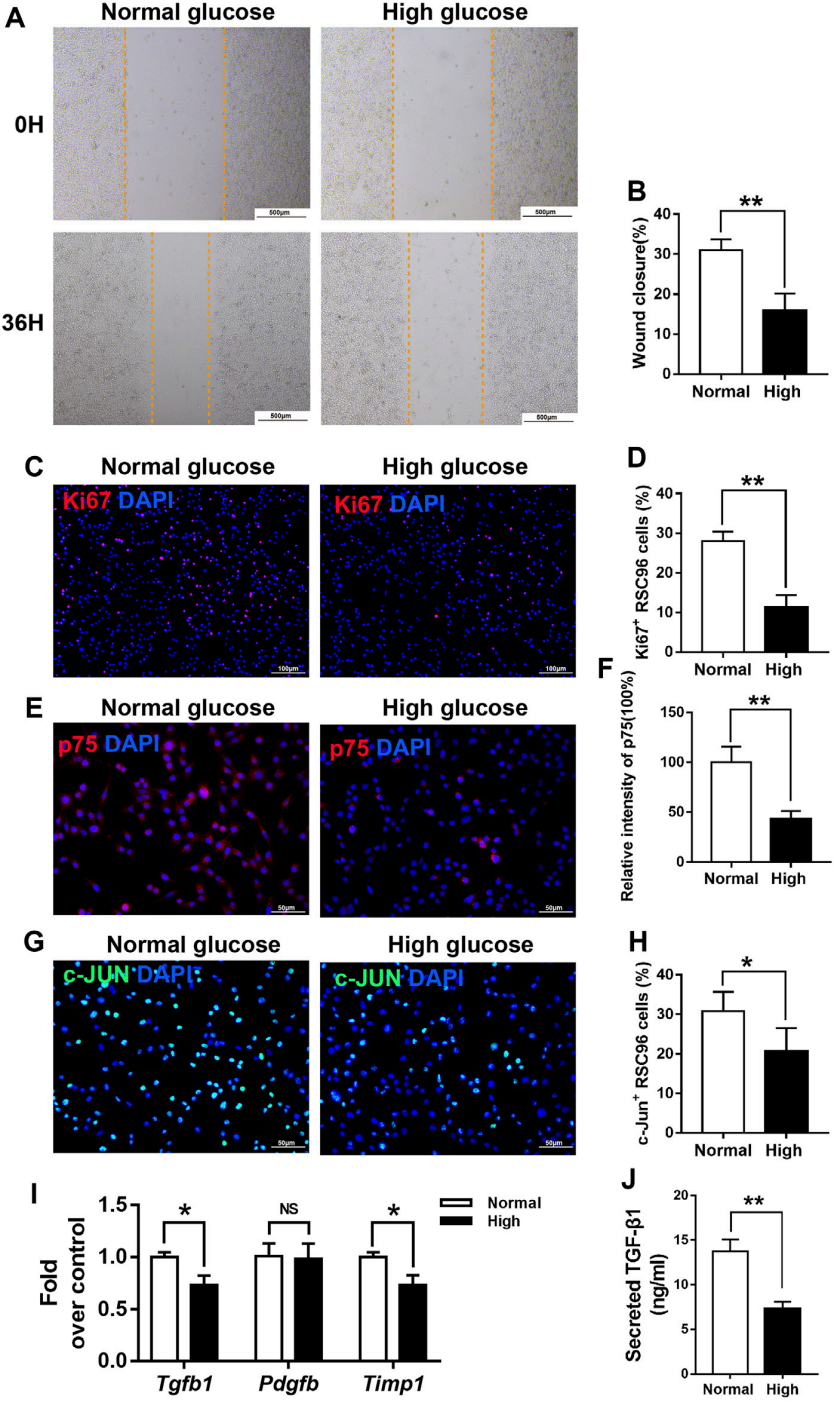
High glucose reduces the viability and de-differentiation of SCs *in vitro*. **(A,B)** A scratch assay of RSC96 cells treated with 5.5 mM glucose (normal) or 50 mM glucose (high) for 0 or 36 h **(C–H)** Representative immunofluorescence analyses of Ki67 **(C)**, p75 **(E)**, and c-Jun **(G)** in cells incubated with normal or high concentrations of glucose for and the percentages of Ki67 + cells **(D)**, p75 + cells **(F)**, and c-Jun + cells **(H)** in the two groups. **(I)** Real-time PCR analyses of the expression levels of *Tgf-β*, *Pdgfb*, and *Timp1* in the normal and high glucose groups, expression levels were normalized to those of *Gapdh*. **(J)** ELISA analyses of the expression levels of TGF-β1 in the normal and high glucose groups. Data are presented as mean ± SD of three independent experiments, **p < .01 and*p < .05, Student’s-test.

Next, we incubated cultured RSC96 cells, a spontaneously transformed rat SC line, with normal (5.5 mM) or high (50 mM) concentrations of glucose to model an important diabetic feature. The effects of 50 mM glucose on cell migration and proliferation were determined using a scratch assay and Ki67 staining. Compared with that of cells incubated under normal glucose condition, the migration of cells treated with 50 mM glucose for 36 h was impaired (Figures 7A, B). In addition, the percentage of Ki67^+^ cells was significantly lower in the high glucose group than in the normal glucose group (Figures 7C, D).

Next, we examined whether hyperglycemia affects SC function and repair responses *in vitro*. As expected, the expression levels of p75 (Figures 7E, F) and c-Jun (Figures 7G, H), markers of the repair cell phenotype, were lower in RSC96 cells cultured in 50 mM glucose for 48 h than in those cultured in 5.5 mM glucose for the same period.

### High glucose decreases the TGF-β1 secretion in SCs

We hypothesized that reduced secretion of pro-fibrotic cytokines caused by delayed de-differentiation of SCs might interrupt efficient wound healing of diabetic skin by reducing both myofibroblast differentiation and the viability of fibroblasts. In support of this idea, we found that the expression levels of the *Tgf-β* and *Timp1* mRNAs were significantly lower in RSC96 cells incubated under high glucose conditions for 48 h than in those incubated under normal glucose conditions for the same period (*p* < .05; Figure 7I). ELISA (*p* < .05; Figure 7J) results also showed that TGF-β1 expression was significantly lower in RSC96 cells incubated under high glucose than in those under normal glucose conditions.

### High glucose attenuates the paracrine effect of SCs on fibroblasts

**FIGURE 8:**
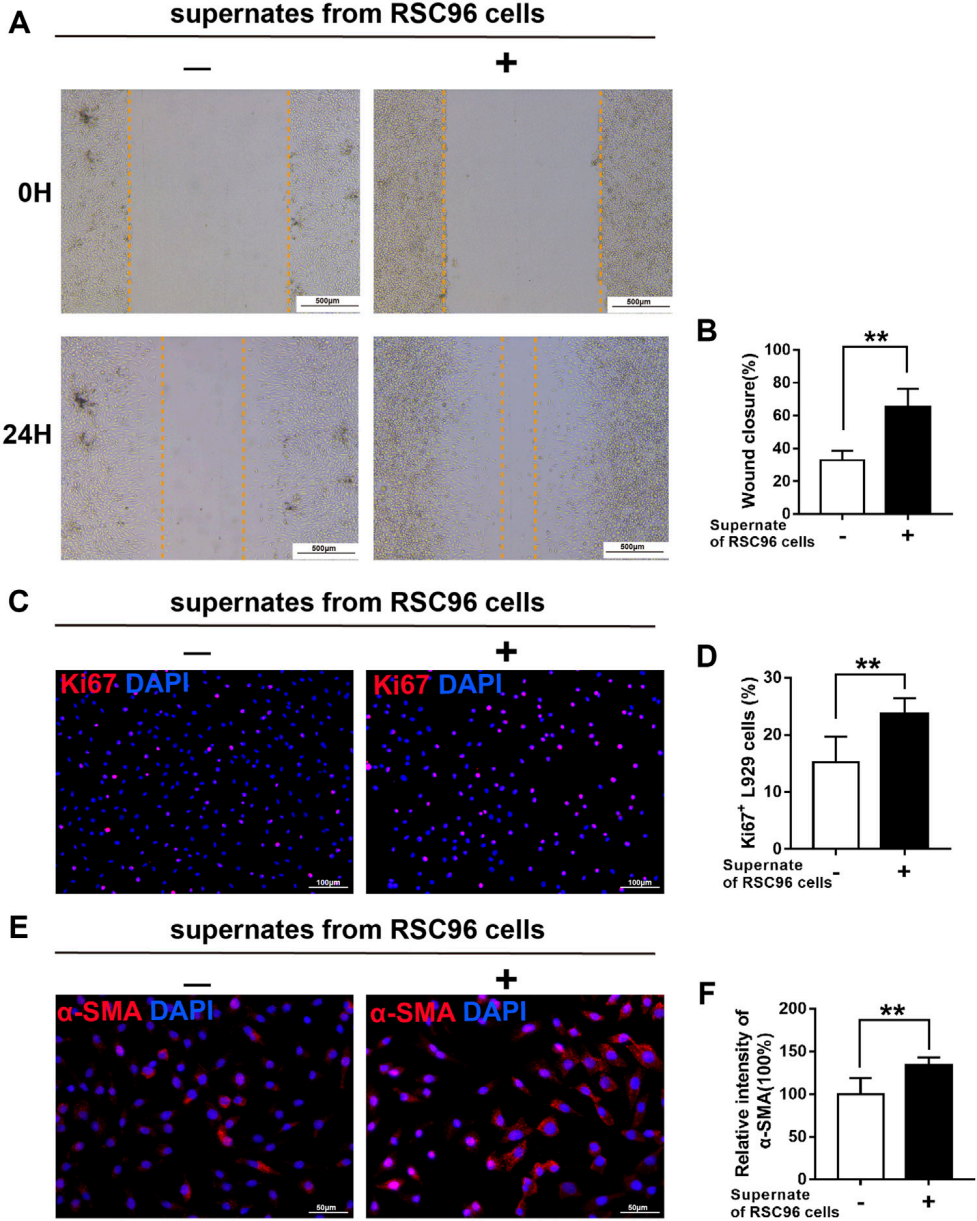
SCs have a paracrine effect on fibroblasts under normal glucose conditions. **(A,B)** A scratch assay of L929 fibroblasts cultured in 5.5 mM glucose and treated for 0 or 24 h with (+) or without (−) supernates from RSC96 cells cultured under the same glucose conditions. Representative immunofluorescence analyses of Ki67 **(C)** and α-SMA **(E)**, and the numbers of Ki67^+^ cells **(D)** and intensity of α-SMA immunoreactivity **(F)**, in L929 fibroblasts cultured and treated as described for **(A,B)**. Data are presented as mean ± SD of three independent experiments, **p < .01, Student’s *t*-test.

**FIGURE 9:**
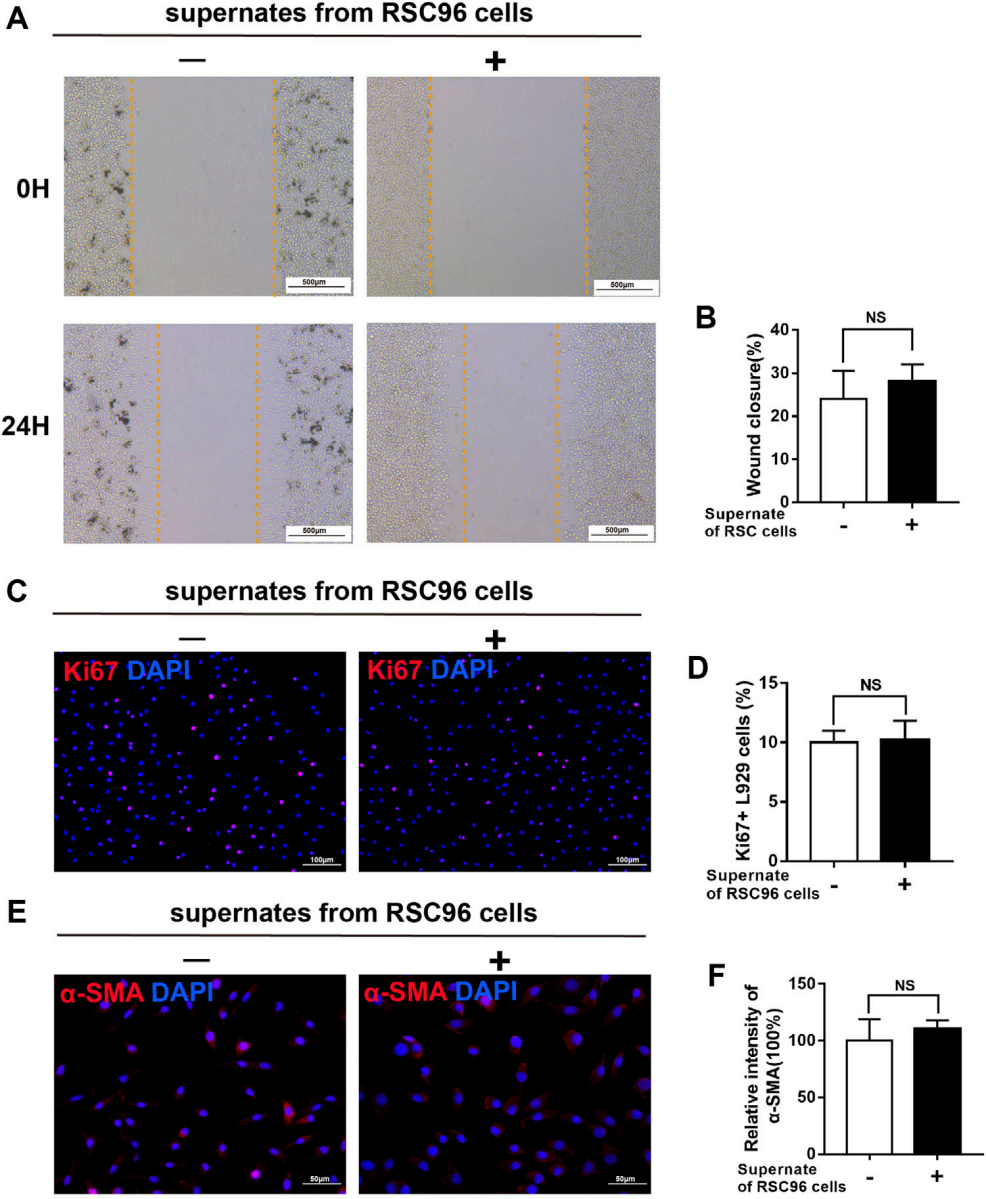
High glucose attenuates the paracrine effect of SCs on fibroblasts. **(A,B)** A scratch assay of L929 fibroblasts cultured in 50 mM glucose and treated for 0 or 24 h with (+) or without (−) supernates from RSC96 cells cultured under the same glucose conditions. Representative immunofluorescence analyses of Ki67 **(C)** and α-SMA **(E)**, and the numbers of Ki67^+^ cells **(D)** and intensity of α-SMA immunoreactivity **(F)**, in L929 fibroblasts cultured and treated as described for **(A,B)**. Data are presented as mean ± SD of three independent experiments, Student’s *t*-test.

Next, we treated L929 fibroblasts with supernates from RSC96 cells cultured under normal (5.5 mM) or high (50 mM) glucose conditions. Compared with L929 cells cultured in 5.5 mM glucose alone, those cultured in 5.5 mM glucose and incubated with supernates from normal glucose-treated RSC96 cells for 24 h displayed enhanced migration (*p* < .05) (Figures 8A, B), increased numbers of Ki67^+^ L929 fibroblasts (*p* < .05) (Figures 8C, D), and increased intensity of α-SMA immunoreactivity (*p* < .05) (Figures 8E, F). By contrast, when L929 cells were incubated with 50 mM glucose and supernates from high glucose-treated RSC96 cells, the levels of cell migration (Figures 9A, B), cell proliferation (Figures 9C, D), and α-SMA immunoreactivity (Figures 9E, F) did not differ significantly from those of cells incubated with 50 mM glucose alone (*p* > .05).

### TGF-β1 treatment is effective for rescuing the viability of fibroblasts under high glucose

**FIGURE 10:**
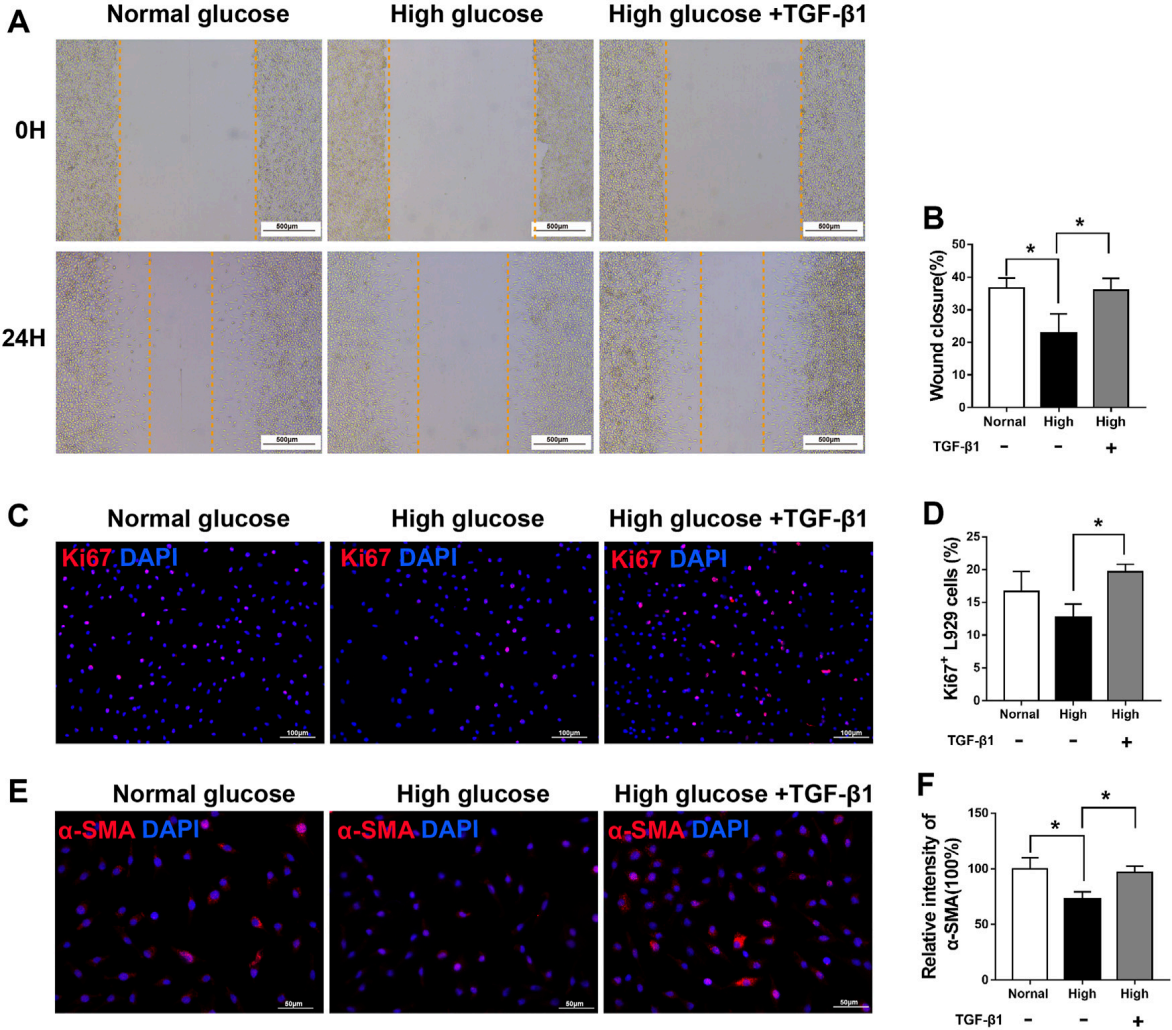
TGF-β1 treatment rescues the viability of fibroblasts under high glucose **(A,B)** A scratch assay of L929 fibroblasts cultured in normal glucose (5.5 mM), high glucose (50 mM) or high glucose added with TGF-β1 (10 ng/ml) conditions. Representative immunofluorescence analyses of Ki67 **(C)** and α-SMA **(E)**, and the numbers of Ki67^+^ cells **(D)** and intensity of α-SMA immunoreactivity **(F)**, in L929 fibroblasts cultured and treated as described for **(A,B)**. Data are presented as mean ± SD of three independent experiments, **p < .01 and*p < .05, one-way ANOVA, Tukey’s post hoc test.

To further test the ability of SCs paracrine factors (especially TGF-β1), we first examined whether hyperglycemia affects fibroblast function *in vitro*. As shown in Figure 10, compared with that of cells incubated under normal glucose conditions, the migration of cells treated with 50 mM glucose for 24 h was impaired. In addition, the α-SMA immunoreactivity was significantly lower in the high glucose group than in the normal glucose group.

However, after TGF-β1 treatment, fibroblasts exhibited improved migration in a scratch migration assay (Figures 10A, B), cell proliferation (Figures 10C, D), and α-SMA immunoreactivity (Figures 10E, F), rescuing the defect in fibroblasts under high glucose conditions.

Those results suggested decreased SCs secreting TGF-β1 may lead to delayed diabetic wound repair.

## Discussion

**FIGURE 11:**
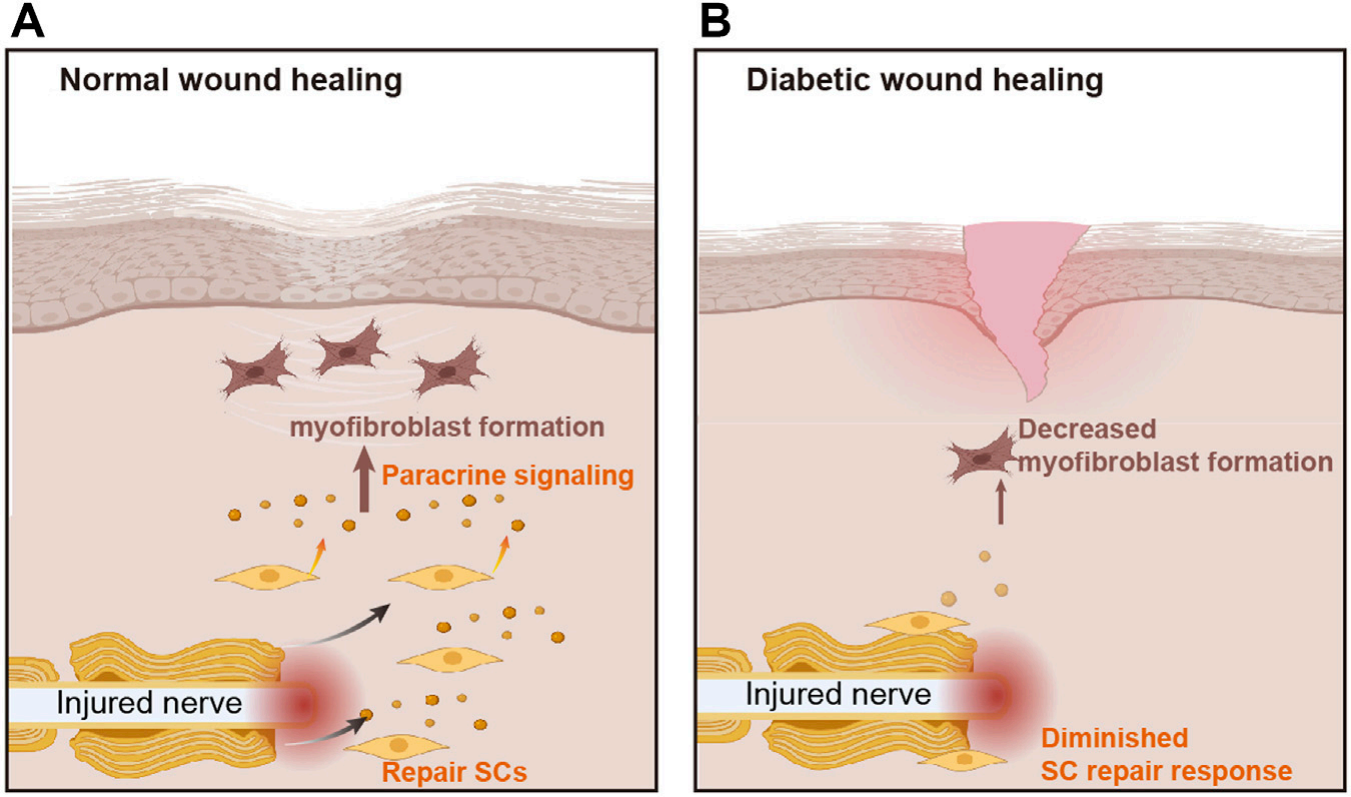
The role of Schwann cells in diabetic wound healing. **(A)** In normal wounds, nerve injury stimulates the aggregation of Schwann cells (SCs), which release paracrine signals to stimulate myofibroblast formation and promote wound healing. **(B)** The repair response of SCs is attenuated in diabetic wounds, resulting in reduced myofibroblast formation and delayed wound healing.

The results presented here highlight the role of diminished SC repair responses in impaired wound healing of the diabetic skin. Using a *db/db* mouse model of excisional cutaneous wounding, we found that diabetic SCs fail to rapidly activate a repair program after injury. Our findings suggest that diabetic SCs display functional impairments in cell de-differentiation, cell-cycle re-entry, and cell wound bed migration. Moreover, we found that a reduction in paracrine TGF-β and Timp1 signaling by SCs under hyperglycemic conditions results in abnormal fibroblast function (Figure 11). Overall, these results suggest that the diabetes-associated attenuation of SC repair responses may lead to slower wound healing in diabetes.

Impaired wound healing is one of the most conspicuous characteristics of DM. The delayed closure of diabetic dermal wounds is accompanied by impaired skin contraction and insufficient fibrous tissue deposition. As demonstrated here, myofibroblast formation, a biological process that accelerates wound healing (Henderson et al., 2020), is also diminished in diabetic wounds. However, the mechanism by which diabetes affects myofibroblast formation is not entirely clear.

This study provides a new view of wound healing pathophysiology, with a focus on neural involvement in normal and diabetic wound healing. Specifically, our results demonstrate that the de-differentiation and repair functions of SCs are impaired in diabetes. It is well known that, after nerve injury, SCs undergo radical change as they adopt phenotypes dedicated to support repair (Painter et al., 2014). SCs are potentially unique among adult differentiated cells in that they undergo a post-injury phenotypic reprogramming process that enables them to acquire a de-differentiated, highly proliferative, almost stem cell-like state (Arthur-Farraj et al., 2012). Recent studies have shown that SCs promote not only the regeneration of nerves, but also that of other tissues, including skin, bone, and the mammalian digit tip (Johnston et al., 2013; Johnston et al., 2016; Jones et al., 2019; Beura et al., 2021). In particular, cells from injured peripheral nerves or from distant sites outside the regenerating dermis have been shown to be involved in skin wound healing (Johnston et al., 2013; Beura et al., 2021). Parfejevs and colleagues demonstrated that SCs contribute to normal adult dermal wound healing by upregulating the expression of TGF-β, which is associated with wound healing and the promotion of myofibroblast differentiation (Parfejevs et al., 2018).

Therefore, we examined whether SCs influence diabetic wound healing. We found that, unlike that in SCs from wounded normal control mice, the expressions of p75 and c-jun, induced markers of the repair cell phenotype, were delayed in SCs at the wound site after skin injury of diabetic mice. Similarly, Pierson *et al.* (Ou et al., 2022)found that the expression of p75 in SCs was delayed and attenuated in diabetic rats after sciatic nerve crush injury, which may contribute to impaired peripheral nerve regeneration. Similar to our study, Ou et al. (2022) found that fewer dedifferentiated SCs recognized by Sox2 expression were observed in the mice with streptozotocin (STZ)-induced diabetes (C57-STZ) wounds than in the C57 wounds, which indicated dysfunction of SC repair responses during diabetic wound healing.

Our analysis demonstrates that SC injury responses are substantially delayed in diabetic wound, a defect we suggest is due to a failure of SCs to effectively activate an appropriate transcriptional repair response after nerve injury under hyperglycemia. Multiple genes are reported to be unregulated, and numerous transcriptional pathways are activated during SC reprogramming. The primary players that control and regulate the repair processes include c-Jun, the mitogen-activated protein kinase (MAPK) pathways, Sonic Hedgehog (Shh), chromatin modifications, Wnt signaling, and the Raf/MEK/ERK signaling pathway (Napoli et al., 2012; Nocera and Jacob, 2020; Ou et al., 2022). A crucial transcription factor in the reprogramming of the SC and the response to peripheral nerve damage is c-Jun. The transcription factor c-Jun was identified as a “master regulator” of the SC repair phenotype after nerve injury; this repair event involves three types of change: 1) Upregulation of repair phenotypes. 2) Downregulation of myelin genes; 3) Activation of stemness genes (Jessen and Mirsky, 2021). Adult nerves have a constitutively low level of expression. By contrast, the expression of the c-Jun gene increases quickly after nerve damage, and it is crucial for axonal regeneration. Indeed, c-Jun cKO mice show delayed demyelination, reduced neuronal survival, and a depressed capacity for regeneration (Hantke et al., 2014). Genetic removal of c-Jun from SCs was reported to result in functionally impaired repair cells and regeneration failure (Huang et al., 2019). In addition, the c-Jun-regulated program represents a portion of the molecular changes induced by injury, which extends to some 4,000–5,000 genes (Jessen and Mirsky, 2021). Most recently, defective c-Jun expression has been linked to the failure of nerve healing during aging and chronic denervation (Wagstaff et al., 2021). We suggest here that the delayed SC responses of diabetic animals may partly result from a failure in an upstream signaling cascade initiated by a change in the injured nerve leading to impaired c-Jun expression. Considering that c-Jun was expressed at lower levels in diabetic mouse wound beds than in normal mouse wound beds, and c-Jun expression, cell de-differentiation, proliferation, and migration were also impaired in SCs under hyperglycemia in our current study. Thus, we supported the hypothesis that hyperglycemia affects the repair response of SCs in part due to decreased c-Jun expression.

The involvement of growth factors or cytokines in the process of diabetic wound healing has been widely explored. The platelet-derived growth factor (PDGF) is a glycoprotein with five dimeric isoforms: PDGF-AA, PDGF-BB, PDGF-AB, PDGF-CC, and PDGF-DD. In the injury area, PDGF-BB is a potent chemokine and mitogen for fibroblasts, keratinocytes, and vascular endothelium (Yang et al., 2020). It also stimulates macrophages to produce and secrete growth factors such as TGF-β (Nishishita and Lin, 2004). Previous research has shown that lentiviral transfection with the PDGF-B gene improves diabetic wound healing, which may be due to its promoting effect on the synthesis of collagen and re-epithelialization (Keswani et al., 2004; Lee et al., 2005). Hence, PDGF-BB is often believed to be effective in wound healing. Treatment with either active protein TIMP-1 or TIMP-1 gene therapy delivered at local wound sites has also been shown to be effective in accelerating diabetic wound healing through its anti-apoptotic effect (Lao et al., 2019). More importantly, a key player in tissue repair is a member of the TGF-β superfamily. TGF-β isoforms have different effects on wound healing. TGF-β3 may facilitate scarless healing in the fetus and reduced scarring in adults, but TGF-β1 may mediate fibrosis in wounds of adults (Lichtman et al., 2016). TGF-β1 is a cytokine involved in wound healing that has been linked to the development of strictures and is thought to be a modulator of fibroblast activation and collagen synthesis. Previous studies have shown that wound healing cytokines are typically secreted by multiple cell types, including macrophages, endothelial cells, and platelets, initiating the cascade leading to scar formation (Frangogiannis, 2020). Thus we mainly explored the different gene or protein expressions of PDGF-BB, TIMP-1, and TGF-β1 between normal and diabetic wound healing.

It is likely that inefficient SC repair responses in diabetic animals have a variety of downstream consequences that impair skin wound healing. The process of tissue repair is accomplished by several growth factors, cytokines, and chemokines in order to deliver messages for cellular migration, proliferation, differentiation, survival, and secretion so as to restore the normal functioning of tissue after injury (Krafts, 2010). Secretion of paracrine factors is one of the main functions of SCs during tissue repair (Solovieva and Bronner, 2021). For instance, SC-derived paracrine factors, including PDGF-AA and oncostatin M, increase the regenerative capacity of the digit tip (Johnston et al., 2016). In addition, SC-derived growth factors are required for skeletal stem cell enactment of bone healing (Jones et al., 2019). Accordingly, we hypothesized that inefficient SC repair responses lead to a reduction in SC-derived paracrine factors during diabetic wound healing. The results presented here indicate that de-differentiated SCs have elevated expression of pro-fibrotic cytokines under normal glucose conditions, but this is impaired under high glucose conditions. Given previous findings that non-myelinating SCs can secrete molecules that regulate latent TGF-β activation (Yamazaki et al., 2011). Here, we also found that reductions in the paracrine levels of TGF-β1, and Timp1 from SCs during hyperglycemia resulted in weakened fibroblast function. These cytokines were selected for analysis on the basis of previous studies describing their roles in SC-dependent healing and skin wound healing (Demaria et al., 2014; Zhou et al., 2021). We suggest that impaired fibroblast activation caused by diminished secretion of paracrine factors by SCs may also contribute to delayed diabetic wound healing, and these paracrine factors are most likely derived from de-differentiated SCs.

This study has some limitations. First, we employed only one line of SCs (RSC96) and fibroblasts (L929) for *in vitro* analyses, experiments using primary cultured cells will need to be formally tested in further studies. Second, our study implicated that the failure of diabetic SCs to efficiently acquire a repair phenotype after nerve injury impinges on diabetic skin wound healing process. In the future, it will be of great interest to determine whether transplanting SCs from normal injured NBs into diabetic wound could improve diabetic wound healing.

In summary, our study links delayed and impaired SC repair responses with diabetic wound healing. Clinically, diabetic wounds are a challenging problem associated with nerve injury and healing complications. Harnessing the underlying biology of nerve-dependent wound healing may hold promise for clinical innovation in diabetic wound healing.

## Data Availability

The original contributions presented in the study are included in the article/supplementary material, further inquiries can be directed to the corresponding authors.

## Abbreviations

DM, diabetes mellitus; MBP, myelin basic protein; NB, nerve bundle; PBS, phosphate-buffered saline; SC, Schwann cells; SMA, smooth muscle actin.
